# Hypertrabeculation in Olympic Athletes: Advanced LV Function Analysis by CMR

**DOI:** 10.3390/jcdd12100388

**Published:** 2025-10-02

**Authors:** Alessandro Spinelli, Sara Monosilio, Giuseppe Di Gioia, Gianni Pedrizzetti, Giovanni Tonti, Cosimo Damiano Daniello, Maria Rosaria Squeo, Antonio Pelliccia, Viviana Maestrini

**Affiliations:** 1 Institute of Sports Medicine and Science, Italian National Olympic Committee, 00197 Rome, Italy; alessandro.spinelli1@gmail.com (A.S.); sara.monosilio@gmail.com (S.M.); antonio.pelliccia@coni.it (A.P.); 2 Department of Clinical, Internal, Anesthesiological and Cardiovascular Sciences, Sapienza University of Rome, 00161 Rome, Italy; 3 Department of Engineering and Architecture, University of Trieste, 34127 Trieste, Italy; 4 Cardiology Division, ‘G. D’Annunzio’ University, 66100 Chieti, Italy

**Keywords:** CMR, hypertrabeculation, strain, hemodynamic forces, athlete’s heart

## Abstract

Left ventricular (LV) hypertrabeculation is increasingly recognized as a phenotype that may reflect physiological adaptation, particularly in athletes exposed to chronic overload, although its functional relevance remains uncertain. This study evaluated the prevalence of excessive trabeculation and its physiological correlation with LV remodeling. We conducted a single-center, cross-sectional study involving 320 Olympic-level athletes without cardiovascular disease. All underwent cardiac magnetic resonance (CMR). Hypertrabeculation was defined by the Petersen criteria. Athletes meeting these criteria were classified as hypertrabeculated and compared with non-hypertrabeculated matched for age, sex, and sport category. LV morphology, function, strain parameters, and hemodynamic forces (HDFs) were analyzed. Hypertrabeculation was identified in 9% of the cohort. No significant differences were observed between groups for training exposure (*p* = 0.262), body surface area (*p* = 0.762), LV volumes (end-diastolic volume indexed *p* = 0.397 end-systolic volume indexed *p* = 0.118), ejection fraction (*p* = 0.101), mass (*p* = 0.919), sphericity index (*p* = 0.419), myocardial wall thickness (*p* = 0.394), tissue characterization (T1 mapping *p* = 0.366, T2 mapping *p* = 0.833), global longitudinal strain (GLS *p* = 0.898), global circumferential strain (GCS *p* = 0.219), or HDFs. All values were within the normal range. In our cohort, LV hypertrabeculation, evaluated by CMR, was relatively common but not associated with structural or functional abnormalities, supporting its interpretation as a benign variant in asymptomatic athletes with normal cardiac function.

## 1. Introduction

Left ventricular (LV) hypertrabeculation represents a morphological trait that varies widely among individuals and may arise as a physiological adaptation in response to increased preload conditions, such as pregnancy and intense physical training, or be correlated to cardiac disease [1,2,3]. While previous classifications introduced the concept of LV noncompaction cardiomyopathy (LVNC), recent evidence disputes the developmental hypothesis underlying this term. Instead, excessive trabeculation or hypertrabeculation is now considered a more appropriate descriptor, especially in the absence of functional abnormalities, fibrosis, or adverse outcome [3].

An increased degree of LV trabeculation has been observed in a non-negligible percentage of athletes (1.4–8.1%), with uncertain clinical implications [2,4]. Whether this finding reflects a benign manifestation of physiological cardiac adaptation, commonly known as the “athlete’s heart”, or underlines the initial stages of a pathological process remains uncertain.

The athlete’s heart is a well-recognized physiological condition characterized by structural and functional myocardial adaptations, including LV mass, enlargement of the LV cavity, augmented wall thickness, and occasionally excessive trabeculation [5]. Therefore, distinguishing physiological from pathological hypertrabeculation is particularly important in sports cardiology, as cardiomyopathies are associated with adverse cardiovascular outcomes, whereas the athlete’s heart is benign [5]. Misclassification may lead to unnecessary restrictions or overlooked risk.

Both echocardiography and cardiac magnetic resonance (CMR) enable the assessment of LV trabeculation. Over the past decades, various diagnostic criteria have been proposed by echocardiography [6,7] and CMR [8,9,10,11]. However, none have been universally recognized as superior or definitive. The criteria proposed by Petersen et al. are the most commonly used in clinical practice by CMR. The criteria are based on the ratio between the non-compacted myocardium and the compacted one measured by CMR with a threshold of >2.3 [11]. Among athletes, the prevalence of excessive trabeculation varies across different disciplines and ethnicities. However, in the absence of systolic dysfunction, the hypertrabeculated phenotype seems to have no prognostic significance. Its association with structural and functional parameters in athletes remains unclear [12]. The increasing use of advanced CMR, such as feature-tracking of myocardial strain and hemodynamic forces (HDFs) analysis, may provide insight into myocardial efficiency and adaptation beyond volumetric parameters or strain alone [13]. HDFs represent the forces exchanged between the blood and the myocardium and are a recognized and validated tool to assess cardiac function nowadays, integrating both the mechanical and the hemodynamic components. They can be calculated by echocardiography and CMR using the cine sequences usually acquired during a standard exam. HDFs have been demonstrated to be altered and misaligned in some pathological conditions, such as heart failure [14].

Previous investigations in athletes with hypertrabeculation have generally reported preserved LV volumes and systolic function [15]. However, there is no data on advanced analysis such as myocardial strain and HDFs in this subgroup.

To address this knowledge gap, the present study aims to determine whether the presence of hypertrabeculation in a cohort of healthy Olympic athletes, as evaluated extensively by CMR, is associated with any abnormality of morphofunctional parameters. By stratifying athletes based on trabecular burden, this research seeks to evaluate the prevalence of excessive trabeculation and its physiological correlation with LV remodeling.

## 2. Methods

The present analysis is part of a cross-sectional and single-center study performed at the Institute of Sports Medicine and Science in Rome. The institute is responsible for the medical evaluation of Italian elite athletes who have been selected for the Olympic Games. A group of 320 Olympic athletes had previously been enrolled in other studies [13,16]. The athletes were free from signs and symptoms of cardiovascular (CV) diseases, without a family history of cardiomyopathies or sudden cardiac death, and with negative results of CV evaluation comprising medical evaluation, standard 12-lead electrocardiogram (ECG), transthoracic echocardiography, and exercise stress test. All athletes voluntarily underwent cardiac magnetic resonance (CMR) without contrast administration, with the intention of establishing CMR standards and characterizing the advanced features of the athlete’s heart. All patients without contraindications to cardiac magnetic resonance were consecutively invited to participate in the study, and none declined participation. Since hypertrabeculation is more prevalent in the Afro-Caribbean population, and they represented only a small percentage of our cohort, we included only Caucasian athletes in our study. Athletes were represented across all sports categories and were classified according to the European Society of Cardiology (ESC) classification [17].

### 2.1. Cardiac Magnetic Resonance

CMR was performed on a 1.5 Tesla scanner (GE Healthcare, Signa^TM^), and cine sequences on the short axis and the three long axes were used for this study. They were obtained using a steady-state free precession sequence acquired in the three long axes and the short axis plane to cover the LV. Specific parameters for the sequences were a slice thickness of 8 mm with a 2 mm gap in each slice, 25 phases per cardiac cycle with a temporal resolution ≤ 45 msec, a repetition time range of 3.2–4.0 msec, views per segment range of 10–12 (set to avoid view-sharing), a matrix of 224 × 200, and a field of view of 350 mm (in-plane resolution 1.56 × 1.75 mm). Short-axis cine images were used to calculate the LV absolute (end-diastolic volume, EDV; end-systolic volume, ESV) and indexed volumes (EDVi; ESVi), mass, ejection fraction (EF), and 3D sphericity index [18,19]. In the evaluation of LV morphological parameters, the papillary muscle and trabeculated layer were included within LV cavity volume.

Strain parameters were computed using dedicated offline software for CMR feature-tracking (*QStrain, Medis bv, Leiden NL, version 1.4*). The LV global longitudinal strain (GLS) and global circumferential strain (GCS) were computed using apical 4-, 2- and 3-chamber cine images [20,21] A semi-automatic region of interest was generated by tracing the endocardial border in systole and diastole from the 4-, 3- and 2-chamber cine images. The software provided LV–GLS and LV–GCS curves, along with their corresponding mean values. LV HDFs were computed using the same software, capturing the interaction between intracavitary blood flow and myocardial wall motion. The analysis was based on input data considering the LV endocardial displacement obtained through strain analysis, as well as estimated areas of the mitral and aortic valve, derived from measurements of the mitral annulus and the left ventricular outflow tract diameters. A 3D reconstruction of the LV endocardial surface permits the calculation of the global HDFs vector by integrating momentum variations across the LV cavity. HDFs were computed during the entire cardiac cycle, in systole and diastole, in their two main directions: apex-to-base (AB) or longitudinal, and lateral-septal (LS) or transversal.

The ratio between the two (LS over AB ratio) was also calculated, representing the HDFs’ vector orientation and providing the overall forces distribution in the LV. HDFs were normalized with LV volumes (to compare ventricles of different sizes) and expressed as a percentage of the force of gravity. Additionally, the root mean square (RMS) of HDFs over time was computed as a dimensionless index of force amplitude. HDF directionality was visualized through polar histograms, and the vector angle was derived to describe the dominant orientation of intraventricular forces over time.

### 2.2. Hypertrabeculation

**Figure 1:**
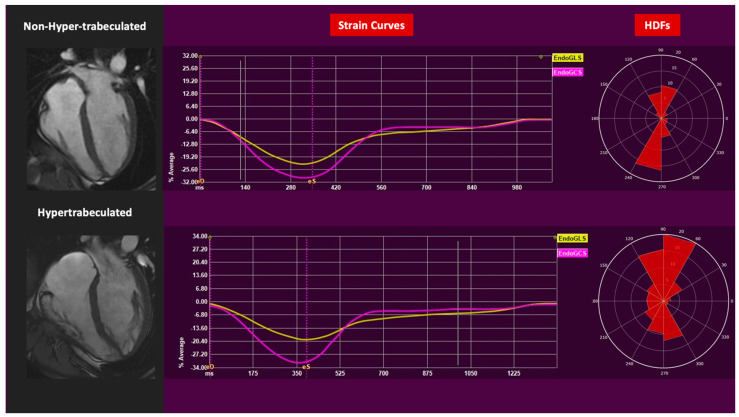
CMR 4 chambers images, GCS, GLS, and HDFs distributions of hypertrabeculated left ventricle and non-hypertrabeculated left ventricle.

Hypertrabeculation (HT) was defined based on the Petersen criteria as the ratio of non-compact (NC) to compact (C) myocardium greater than 2.3 (NC/C > 2.3) [11], measured in apical projections at end-diastole. To determine which athletes met Petersen’s criteria, we selected the segment with the most prominent myocardial trabeculations in the three diastolic longitudinal axes to quantify the thickness of non-compacted (NC) and compacted myocardium (C), with measurements made perpendicular to the compacted wall (Figure 1). We determined the ratio between non-compacted and compacted myocardium thickness (NC/C ratio) in the diastolic phase for each of the three longitudinal projections. We considered only the maximum value of this ratio for analysis. We omitted measurements from the apical segment because, in this region, the compacted myocardium tends to have reduced thickness, which could potentially not be representative of NC/C ratio values. In the overall cohort, n = 30 athletes (9%) met the criteria for hypertrabeculation. Among the remaining 290 athletes, 30 without hypertrabeculation (non-hypertrabeculated, NHT) were matched according to age, sex, and sports categories, and were selected to compare morphofunctional characteristics between the two groups (HT versus NHT).

### 2.3. Statistical Analysis

We performed statistical analysis using the Statistical Package for Social Sciences, version 27.0 (SPSS, Chicago, IL, USA). We tested continuous variables for normality distribution and expressed them as mean and standard deviation or median between 25th and 75th percentiles when they did not show a normal distribution. We presented categorical variables as numbers and percentages. We used Student’s *t*-test or the Mann–Whitney test for continuous variables, and the χ2 test or Fisher’s exact test for categorical variables, when appropriate.

We set the statistical significance level at *p* < 0.05.

## 3. Results

Of the overall cohort of 320 Olympic athletes, 30 (9%) represented the group of athletes with HT. They were compared with 30 NHT athletes who matched their age, sex, and sports categories (power 23.3%, mixed 26.6%, and endurance 50%) as shown in Table 1.

There were no significant differences in the amount of training between the two groups, in terms of years (YT) and hours of training per week (HTW) between NHT and HT, respectively (YT: 16 ± 6 vs. 13 ± 5, *p* = 0.065; HTW: 26 ± 11 vs. 30 ± 11, *p* = 0.262), nor in their performance at the cycle ergometer test in terms of peak workload (NHT: 3.4 ± 0.6 vs. 3.5 ± 0.7, *p* = 0.771).

The morphofunctional analysis did not reveal any significant differences. Indeed, comparisons between NHT and HT for the EDVi (108 ± 19 vs. 113 ± 16, *p* = 0.397), ESVi (47 ± 9 vs. 51 ± 9, *p* = 0.118), maximum LV wall thickness (10 ± 1 vs. 9 ± 1, *p* = 0.394), mass (64 ± 16 vs. 64 ± 19, *p* = 0.919), and the sphericity index (43 ± 6 vs. 42 ± 8, *p* = 0.419) do not yield significant differences.

Mapping analysis did not reveal any significant differences in either T2 mapping values or native T1 mapping (T2 mapping, *p* = 0.833; T1 mapping, *p* = 0.366). The same was found for advanced functional analysis. Specifically, GLS and GCS showed no significant differences between NHT and HT, respectively (GLS: −22 ± 3 vs. −22 ± 3, *p* = 0.898; GCS: −31 ± 4 vs. −29 ± 3, *p* = 0.219). HDFs did not exhibit significant differences either. None of the HDFs components measured in the entire cardiac cycle, in systole and diastole, differed between the two groups (Table 1). Moreover, none of the athletes selected in the examined cohort reported any symptoms or showed any signs of cardiovascular pathology, as determined by clinical and instrumental evaluation.

## 4. Discussion

Clinicians and scientists have long investigated the significant changes the heart undergoes due to intense athletic training. Among these adaptations, dilatation of cardiac chambers and myocardial wall hypertrophy have been well-documented [5,22,23]. In recent years, there has been a significant conceptual shift in the interpretation of pronounced LV trabeculation, diverging from its traditional association with LVNC towards a more refined and context-dependent understanding of increased trabecular patterns. Excessive trabeculation, particularly in the context of healthy individuals, including athletes, or women during pregnancy, is increasingly considered a likely benign anatomical variant or physiological adaptation to volume load, rather than a cardiomyopathy [5].

In our cohort of 320 elite Olympic athletes, we identified a 9% prevalence of hypertrabeculation based on the widely adopted Petersen criteria. This finding aligns with previous studies in the general population and among athletes, which report a variable prevalence ranging from 1.4% to 8%, depending on the imaging modality, diagnostic criteria, and population characteristics. Of note, when comparing athletes with and without hypertrabeculation, matched according to age, sex and sport type, we did not observe significant differences in LV volumetric indices, myocardial mass, global strain parameters, or novel indices such as HDFs.

These findings are clinically relevant for several reasons. First, they reinforce the concept that, in the absence of systolic dysfunction or other structural abnormalities, hypertrabeculation in athletes may represent a non-pathological expression of cardiac remodeling. Indeed, consistent with the recent ESC guidelines on cardiomyopathies, excessive trabeculation should not be considered an independent disease entity unless accompanied by functional impairment, myocardial fibrosis, or adverse outcomes [12,24].

Second, our data provide further evidence that hypertrabeculation is a benign morphological variant and does not correlate with impaired myocardial performance. No significant differences in LV GLS, GCS, or HDFs orientation and magnitude were found. The measured GLS and GCS values indicate that myocardial hypertrabeculation in athletes does not compromise global myocardial deformation, as these parameters remain within physiological ranges and do not differ significantly from those observed in athletes without hypertrabeculation. Additionally, the absence of significant differences in HDF parameters further highlights the interpretation of hypertrabeculation as a benign, non-pathological adaptation in this population. The capability of HDFs to differentiate physiological and pathological conditions, such as dilated cardiomyopathy, heart failure, or myocardial infarction, has been previously demonstrated. Indeed, in such conditions, a misalignment of the HDFs could be observed, meaning that LS-directed or transversal forces tend to predominate over the AB or longitudinal ones [14,25]. This finding aligns with previous studies, which have shown that strain imaging does not indicate any detrimental effects associated with trabeculation in isolation or as a result of sport participation [13,14].

Third, this study adds to the ongoing effort to refine diagnostic thresholds in the assessment of athletes. Reliance on rigid morphological thresholds, such as NC/C > 2.3, has raised concerns regarding potential overdiagnosis of LVNC or “noncompaction-like” phenotypes in healthy individuals. Our findings suggest that excessive trabeculation in athletes, even when meeting established diagnostic criteria, does not necessarily indicate functional compromise or underlying pathology. These observations are consistent with emerging recommendations that emphasize a more integrative approach to morphological findings, taking into account clinical history, performance level, and functional imaging data. It further reinforces the concept that the hypertrabeculated phenotype should not be considered pathological in the absence of other clinical and instrumental evidence of disease, since it may be observed even in a cohort of healthy, asymptomatic athletes.

Interestingly, although some volumetric parameters (EDVi, ESVi, LV mass) tended to be slightly higher in the hypertrabeculated group, these differences did not reach statistical significance and were well within the physiological range. These differences, albeit minimal, could be determined by our methodological approach of including the trabeculated layer within the LV cavity volume, although this was also conducted for NHT group that was not completely free of myocardial trabeculae. No differences were observed in non-contrast tissue characterization (native T1 and T2 mapping), suggesting that myocardial composition is preserved regardless of trabecular burden. Similarly, the sphericity index and maximum wall thickness were comparable across groups, further supporting the absence of pathological remodeling.

Taken together, these results suggest that in highly trained athletes, hypertrabeculation as defined by CMR does not carry negative implications for myocardial function, structure, or tissue integrity. This reinforces the clinical principle that, in asymptomatic individuals with normal systolic function and no family history or electrocardiographic abnormalities, excessive trabeculation should not prompt restrictive decisions regarding sports participation or require further investigations.

## 5. Limits

Limitations of our study include its cross-sectional design and single-center nature, which may impact causal inference and generalizability. Although our population is highly homogeneous and well-phenotyped, longitudinal studies are necessary to determine whether a subset of hyper trabeculated athletes may develop functional impairment over time. Moreover, individuals of Afro-Caribbean ethnicity are not represented. We also acknowledge that no contrast agent was used during imaging, thus complete tissue characterization was not performed. However, pre-contrast tissue characterization through T1 and T2 mapping was performed and had normal results. Another limitation is due to our methodological approach to include trabeculated myocardium and papillary muscles within the LV cavity volume, as applied in some CMR studies. This protocol may introduce systematic biases in the presence of pronounced trabeculation such as overestimation of EDV and ESV and underestimation of the LV mass [26]. Finally, athletes with known cardiomyopathies were excluded from the study, which may limit the applicability of our findings to populations with early or subclinical myocardial disease.

## 6. Conclusions

In conclusion, our study contributes to the growing body of evidence that LV hypetrabeculation in elite athletes is not a rare phenomenon (involving 9% of the subjects) and represents a benign anatomical variant without functional consequences. These findings support a conservative approach to the clinical management of trabecular patterns in the athletic heart and call for updated interpretive frameworks that move beyond rigid morphological thresholds. These findings support the idea that an increased degree of trabeculation, in the absence of other structural or functional abnormalities, should not be considered a sufficient indication for further diagnostic testing or lifestyle restrictions, including sport participation. Moreover, advanced CMR parameters for evaluating cardiac function, such as strain and HDFs, show potential as new tools for improving diagnostic accuracy in the evaluation of elite athletes.

## Figures and Tables

**Table 1:** Comparison between hypertrabeculated and non-hypertrabeculated athletes.

Parameters	Non-Hypertrabeculated n = 30	Hypertrabeculated n = 30	*p* Value
**Age, years**	25 ± 5	24 ± 4	0.377
**Years of training, years**	16 ± 6	13 ± 5	0.065
**Hours of training per week, h**	26 ± 11	30 ± 11	0.262
**Sports categories**	Power (23.3% n = 7), Mixed (26.6% n = 8), Endurance (50% n = 15)	Power (23.3% n = 7), Mixed (26.6% n = 8), Endurance (50% n = 15)	N.A.
**BSA, m^2^**	2 ± 0.2	2 ± 0.3	0.762
**Workload peak, Watt/kg**	3.4 ± 0.6	3.5 ± 0.7	0.771
**EDV[i], mL/BSA**	108 ± 19	113 ± 16	0.397
**ESV[i], mL/BSA**	47 ± 9	51 ± 9	0.118
**EF, %**	57 ± 4	55 ± 4	0.101
**Mass[i], g/BSA**	64 ± 16	64 ± 19	0.919
**LVMWT max, mm**	10 ± 1	9 ± 1	0.394
**Sphericity index, %**	43 ± 6	42 ± 8	0.419
**T2 mapping, ms**	51 ± 5	52 ± 3	0.833
**T2 weighted for edema sequences**	0%	0%	N.A.
**T1 native myocardial mapping, ms**	941 ± 26	945 ± 24	0.366
**T1 weighted for fat sequences**	0%	0%	N.A.
**GLS, %**	−22 ± 3	−22 ± 3	0.898
**GCS, %**	−31 ± 4	−29 ± 3	0.219
**HDF ratio entire, %**	14 (13–16)	14 (12–17)	0.969
**HDF ratio systole, %**	11 (9–14)	11 (9–13)	0.474
**HDF ratio diastole, %**	22 (19–29)	22 (19–26)	0.846
**HDF AB entire %**	21 (18–26)	20 (19–23)	0.465
**HDF AB systole %**	35 (30–40)	32 (29–35)	0.092
**HDF AB diastole %**	11 (9–13)	12 (9–13)	0.592
**HDF LS entire %**	3 (2–4)	3 (2–4)	0.437
**HDF LS systole %**	4 (3–5)	3 (3–4)	0.072
**HDF LS diastole %**	3 (2–3)	3 (2–3)	0.347

**Abbreviations.** BSA: body surface area; EDVi: end-diastolic volume index; ESVi: end-systolic volume index; EF: ejection fraction; LVMWT: left ventricle max wall thickness; GLS: global longitudinal strain; GLC: global circumferential strain; HDFs: hemodynamic forces; AB: apical–basal; LS: lateral-septal.

## Data Availability

The data presented in this study are available on request from the corresponding author, without undue reservation.
